# Exploring Non-invasive Therapies for Bell’s Palsy: A Case Report

**DOI:** 10.7759/cureus.63071

**Published:** 2024-06-24

**Authors:** Prem A Sawarbandhe, Swapnil Mohod, Mahek R Batra, Arshjot S Basra

**Affiliations:** 1 Oral Medicine and Radiology, Sharad Pawar Dental College and Hospital, Datta Meghe Institute of Higher Education and Research, Wardha, IND

**Keywords:** facial nerve paralysis, corticosteroid treatment, diagnosis and management of facial pain, facial asymmetry, bells palsy

## Abstract

Unknown in origin, Bell's palsy is a common acute facial nerve paralysis that is usually characterized by unilateral facial weakening or paralysis. People of all ages are affected by this illness, which peaks in the fourth decade of life. Although the precise etiology is yet unknown, viral infections - particularly type 1 herpes simplex virus - are frequently linked to the problem. Based on the evidence of abrupt onset facial weakness and the elimination of other neurological diseases, the diagnosis is essentially clinical. The goals of management techniques are to lessen related symptoms, encourage nerve regeneration, and lessen inflammation. Corticosteroids, antiviral drugs, physical therapy, and supportive measures are available as treatment alternatives. The majority of patients experience spontaneous recovery within weeks to months, and the prognosis is generally excellent. Nonetheless, a portion may experience long-term consequences, highlighting the significance of individualized follow-up care. Bell's palsy is succinctly summarized in this abstract to aid in better comprehension and well-informed clinical practice decision-making.

## Introduction

Acute unilateral facial paralysis most frequently results from Bell's palsy, named after Sir Charles Bell, who first characterized the illness in the early 1800s. This disorder, which is defined by the sudden onset of paralysis or muscle weakness on one side of the face, is caused by the seventh cranial nerve, commonly known as the facial nerve. A person's identity and personality are greatly influenced by the traits on their face. In addition to physical handicap, a loss in facial muscle function causes social and psychological discomfort since facial expressions are essential for expressing emotions and interacting with people [1]. Between 60% and 75% of all cases of facial paralysis are linked to Bell's palsy. There are seven to 40 instances annually per 100,000 people, with the same prevalence rates for males and women. Hyperacusis, dysgeusia, subjective changes in facial response, and postauricular discomfort are examples of concurrent symptoms [2]. While there is still no clear cause for Bell's palsy, a number of reasonable theories have been proposed. These comprise infections, genetic syndromes, herpes simplex virus, traumatic events, congenital causes, birth trauma, osteopetrosis, Melkerson-Rosenthal syndrome, oculoauriculovertebral dysplasia, and conditions associated with cancer [3]. Clinically, Bell's palsy patients usually experience a rapid onset of unilateral facial paralysis that peaks in 48 hours. Patients may have the following symptoms: flattening of the creases on the affected side of their foreheads, drooping of the mouth, trouble closing their eyes, and disappearance of the nasolabial fold. Reduced tearing, hyperacusis (increased sensitivity to sound), and changed taste perception are possible adverse effects. Patients may occasionally complain of ear pain prior to the development of facial paralysis. The primary physical examination finding is a weakening of the forehead, either whole or partial. If the forehead's strength is maintained, a central cause needs to be investigated. Patients may also report fluctuations in salivation and tears, taste alterations, otalgia, and sound sensitivity. Ocular characteristics include things like corneal exposure, brow droop, loss of the nasolabial fold, upper eyelid retraction, paralytic ectropion of the lower lid, lagophthalmos, and more. Bell's palsy is often diagnosed clinically after all other possible causes are eliminated and the distinctive appearance of abrupt unilateral facial paralysis is noted. Examples of differential diagnosis include Ramsay Hunt syndrome, otitis media, malignancy, and stroke. CT scans and MRIs are two common imaging tests used to rule out these additional conditions. It may be necessary to perform laboratory testing to look for infections or other underlying issues. Management strategies aim to lessen related symptoms, encourage nerve regeneration, and reduce inflammation. Other treatment options include corticosteroids, antiviral medications, physical therapy, and supportive measures [4].

## Case presentation

**Figure 1:**
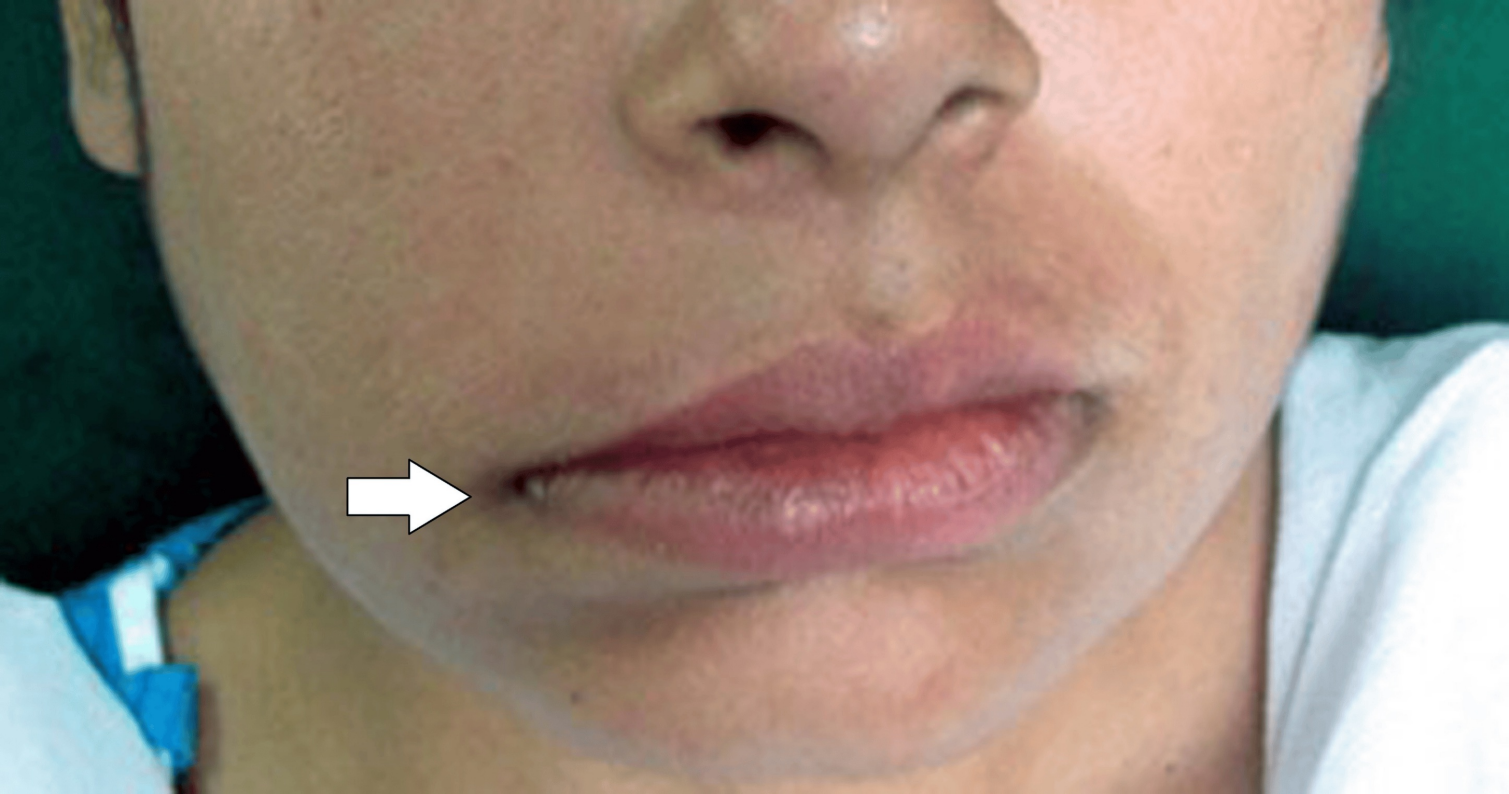
Extraoral examination of the patient showing asymmetrical face and drooping of the right corner of the mouth

A 28-year-old woman with a history of facial asymmetry since childhood came to the oral medicine department of a tertiary care facility in Sawangi, Wardha, India. The other complaints were jaw pain and issues with speaking; the patient was not on any medications. The patient presented with no history of trauma, negative family history, significant medical history, or dental history. On extraoral examination, there was noticeable bilateral facial asymmetry. Upon examination, the temporomandibular joints (TMJ) were found to be bilaterally smooth and synchronous, indicating no irregularities in TMJ function. However, the patient exhibited an inability to close the right eye, a clinical indication consistent with Bell’s sign. Additionally, there was a drooping of the right corner of the mouth, as shown in Figure 1. The intraoral examination revealed multiple dental and periodontal issues. In the lower right quadrant, the first molar was grossly decayed, while pit and fissure caries were observed on the third molar of the same quadrant, and buccal pit caries were present on the second molar. The examination also noted the absence of the first molar in the lower left region of the jaw. Generalized gingival inflammation was evident throughout the mouth, indicating a widespread periodontal condition. Additionally, the patient exhibited an anterior crossbite. Significant staining and the presence of substantial calculus were also noted.

**Figure 2:**
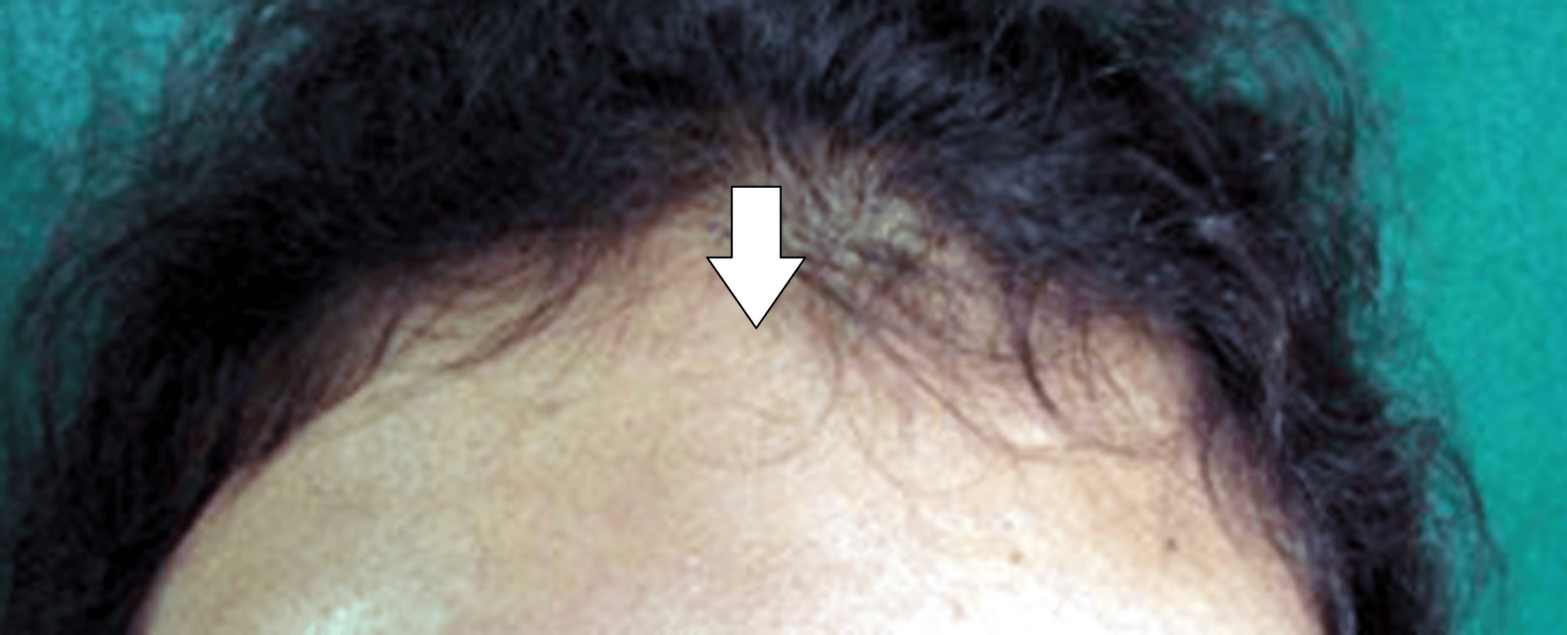
Patient's forehead appears free from wrinkles

Upon closer inspection, it was discovered that the patient was unable to move their lips, lift their mouth corner, open or close their eyes, or display additional symptoms like dry eyes, ipsilateral ear pain or hearing loss, nasolabial fold flattening, ipsilateral brow sagging, or mouth drooping. The forehead's ipsilateral side is wrinkle-free when the eyebrows are raised, as seen in Figure 2.

**Figure 3:**
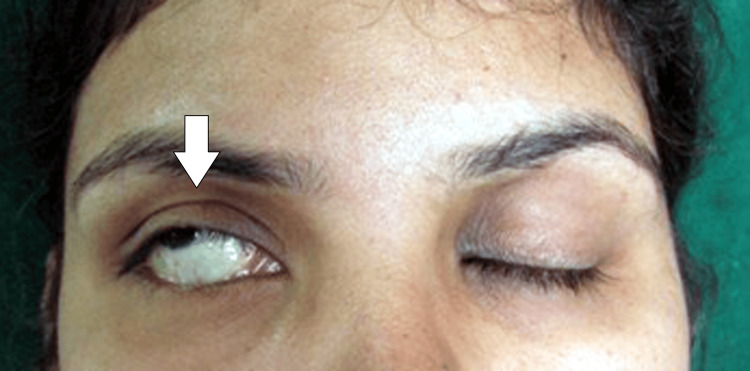
Patient shows positive Bell’s sign

The patient's ipsilateral eye displays incomplete closure and may remain slightly open when she tries to close his eyes. Additionally, the damaged eyelid may blink a tad more slowly. The patient's eyes deviated laterally and upward when asked to close their eyelids tightly, as seen in Figure 3.

**Figure 4:**
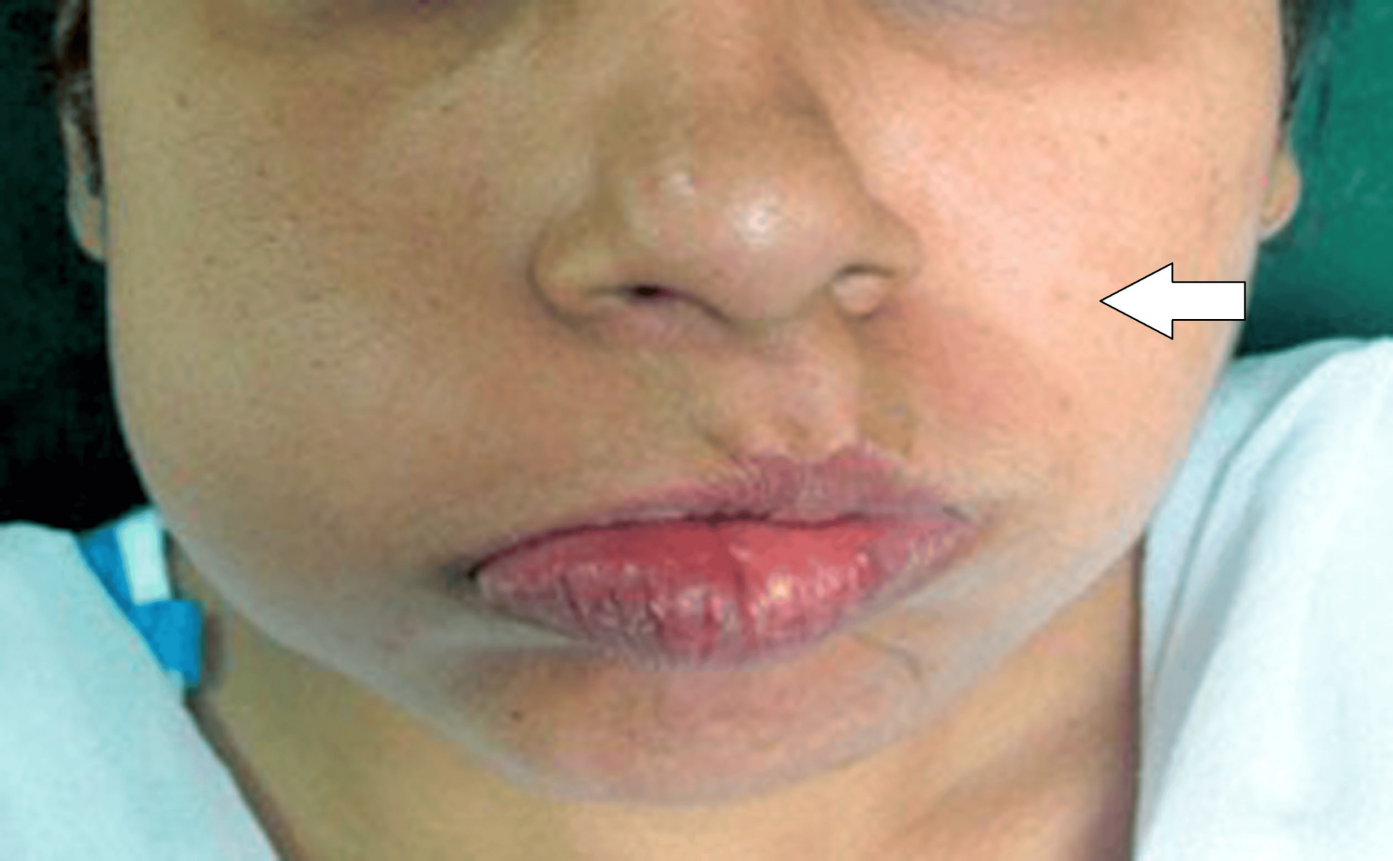
Inability to blow cheek on left side

The patient was unable to close their eyelids and attempted to open them instead. Bell's phenomenon is an eye defense mechanism that manifests when someone closes their eyelids firmly. Rather than shutting entirely, the eyeballs move in an upward and outward rotation. When the eyelids close firmly, as they do when blinking or closing the eyes tightly, this movement helps shield the cornea and the front of the eye from harm. When the patient was asked to blow the cheek, she was unable to blow on the left side, as shown in Figure 4.

The patient was diagnosed with congenital Bell's palsy affecting the right side of the face based on clinical evidence. The patient received emotional and mental assistance regarding her condition through counseling after being informed about it. The treatment was made clear, and the treatment plan was prescribed. Following the patient's initial period of close observation, routine follow-up appointments were set up to track the patient's general health and performance of the facial muscles. Physical therapy was used as an early intervention to enhance muscular tone and function. A program of face workouts designed to activate the afflicted muscles and encourage symmetry was part of the therapy strategy. Daily performance of these activities was supervised by a physical therapist. This training promoted consistent muscle use and growth and ensured the continuation of therapy outside of clinical settings. Because the left eyelid could not be closed all the way, precautionary measures were taken to avoid eye injury. Several times a day, lubricating eye drops were given four times a day (QID), and at night, an ointment was applied twice a day (BD) to keep the cornea wet and protect it. However, if a noticeable improvement was not shown over time, the patient was scheduled for frequent evaluations to see whether additional treatments like nerve grafting or muscle transposition surgery would be necessary. The patient received a well-rounded strategy to aid in healing and enhance quality of life through this extensive treatment plan. Frequent follow-up made it possible to modify the treatment strategy in light of the patient's development. She had no problems speaking, no jaw pain, and no facial drooping during repose after two months of therapy. She was advised to keep doing the facial muscle exercises until her baseline function returned at this point.

## Discussion

Sir Charles Bell originally defined Bell's palsy in 1821. It is an acute-onset idiopathic facial paralysis caused by a failure somewhere along the peripheral part of the facial nerve, starting at the distal level of the pons. The precise etiology of Bell's palsy is unknown, but several explanations have been proposed, including venous congestion or ischemia, a virus resembling zoster or herpes simplex, arteriospasm-induced physiologic compression of the nerve, and restriction of the bone canal. Several case studies reflect a familial tendency suggesting the transmission of an aberrant facial canal [5]. The ability to communicate with their faces is crucial for an individual's ability to feel good about themselves and integrate into social networks. Because of this, those who have facial palsy may go through a very difficult psychological period. The combination of antiviral medications and steroids in the treatment of Bell’s palsy may somewhat improve the prognosis, while the effectiveness of facial nerve decompression as a Bell’s palsy therapy strategy is still under question [6]. Bell's phenomenon describes how the lower lid sags and the eye rolls upward as one tries to close the eyelids. Both prolonged exposure and dehydration might result in ocular irritation. Although the eye can no longer cover its lids tightly, tears may appear to be coming out of it excessively even though the production of tears decreases. Although they are paralyzed, patients often describe feeling numb [7]. However, they are still able to feel their faces. The purpose of treatment is to expedite the healing process and prevent corneal issues. Proper eye care involves not just patching and lubricating the eyes but also using lubricating drops often during the day and an ointment at night. The majority of patients usually have a good prognosis. The majority of individuals fully recover; about 13% have mild paresis, and 4-5% have severe facial impairment, according to The Copenhagen Facial Nerve Study [8]. Even if medicinal interventions help patients feel better faster and with fewer symptoms, the effectiveness of these interventions is still up for debate because most people recover on their own. Reducing neuronal damage and enhancing the function of the seventh cranial nerve, or the face nerve, are the objectives of treatment. Bell's palsy should be treated conservatively, taking into account the severity and likely prognosis of each individual case. Although protecting the eyes is so important, lubricants and eye patches are used to keep the cornea from drying out. During the day, use lubricating eye drops like hypromellose drops, and at night, apply ointment. In extreme situations, the eye might need to be partially or completely sutured shut. The treatment's main objectives are complex. First and foremost, the goal is to improve facial nerve function in order to restore the control and expression of the facial muscles. The second goal is to reduce the amount of damage to neurons by using therapeutic interventions to stop the degeneration of brain tissues from getting worse. Preventing problems resulting from corneal exposure is also a crucial part of the treatment plan, as it protects the integrity and overall health of the eye. Through a complete approach to these objectives, the treatment aims to maximize functional and aesthetic results while putting the patient's general health first. The treatment approaches are drug therapy, surgical therapy, and physical therapy.

Drug therapy

For Bell's palsy, oral corticosteroids (such as prednisolone) and antibiotics are often used treatments. It is established that swelling and edema in the facial nerve are the cause of the symptoms. Corticosteroids are therefore employed because of their anti-inflammatory properties [9].

Corticosteroids

Because corticosteroids have strong anti-inflammatory properties, such as prednisone, they are regarded as the first-line treatment. Prednisone is normally started on a high dose for the first few days of the regimen, often 60-80 mg per day, and then tapered off gradually over the period of one to two weeks. Efficient delivery is essential to maximize the chance of full recovery, preferably within 72 hours after the onset of symptoms. Corticosteroids aid in lowering edema and inflammation around nerves, which lessens nerve compression and improves functional recovery. Clinical studies have consistently shown that patients receiving corticosteroids have better outcomes in terms of facial nerve function compared to those who do not receive this treatment. However, it is important to monitor for potential side effects, including hyperglycemia, hypertension, and increased susceptibility to infections.

Antiviral Drugs

As the herpes simplex virus may be the cause of the ailment, antiviral medications have been investigated as potential treatments. Corticosteroids and antiviral drugs like valacyclovir or acyclovir are frequently administered together. Targeting and blocking viral replication is the idea of antiviral medication, which may lessen viral load and consequent nerve damage. Typically, an antiviral treatment consists of either 400 mg of acyclovir five times a day or 1,000 mg of valacyclovir three times a day for seven to 10 days. Antiviral medication by itself is not proven to be very successful; however, when paired with corticosteroids, there is some indication of improved outcomes, particularly in severe cases of Bell's palsy. Nonetheless, the advantages of combining antivirals with corticosteroid therapy are still being investigated, and because of the conflicting data, certain clinical guidelines do not always support this pairing.

Bell's palsy late sequelae are probably less common when antivirals and corticosteroids are used in combination rather than when corticosteroids are used alone. Research has also demonstrated that individuals using corticosteroids had a lower incidence of long-term side effects compared to those receiving antivirals [10].

Physical therapy

Patients with Bell's palsy, a disorder characterized by abrupt, unilateral facial nerve paralysis, benefit greatly from physical therapy. Physical therapy aims to prevent long-term issues, including muscle contractures and synkinesis (involuntary movements), and to improve the strength, coordination, and function of the facial muscles. Bell's physical therapy for cerebral palsy involves various essential components focused on improving muscle strength, nerve regeneration, and overall facial function. Face exercises are central, targeting specific facial muscles with repetitive movements like frowning, smiling, and eyebrow-raising. Additionally, rehabilitative massages are utilized to prevent muscle contractures, enhance blood flow, and alleviate stiffness, thereby enhancing flexibility and relaxation. Biofeedback provides immediate feedback to help patients control facial muscles and reduce involuntary movements. While the effectiveness of low-level electrical stimulation is still uncertain, supervised application may aid muscle activation and prevent atrophy. Mirror treatment allows patients to observe and change their facial motions visually, enhancing symmetry and control. Eye care advice is also provided to prevent dryness and corneal damage, given the potential impact of Bell's palsy on eye closure. Counseling and emotional support are integral in providing education about the condition, recovery expectations, and the importance of consistent therapy. As physical therapy becomes increasingly common for facial paralysis, personalized strategies can significantly improve recovery prospects [11]. Without treatment, 71% of Bell's palsy patients fully restore their motor function in less than six months. All Bell's palsy patients ought to have made some progress by the end of six months. Among the poor prognostic variables include advanced age, high blood pressure, diabetes, taste impairment, and whole facial paralysis [12].

## Conclusions

We now know more about Bell's palsy and can better understand its causes, symptoms, and available treatments. As a result of a comprehensive analysis of patient data and evaluation of clinical results, we determined that this disease is affected by many factors. These include genetic factors, weak immunity, and infections. Additionally, to improve overall patient outcomes, our study highlights the urgent need for early diagnosis and treatment such as corticosteroid therapy and physical therapy. Although we have come a long way in understanding and treating Bell's palsy, there are still some issues to solve. Patients' response to treatment varies, reflecting the value of individual opinion, including variability, treatment preferences, and disease severity. In addition, it is necessary to investigate the long-term effects of Bell's palsy, such as facial asymmetry and functional impairment, to develop specific solutions to these problems, improve quality of life and reduce mental health problems. Our research contributes significantly to the growing body of knowledge and aims to improve our understanding and treatment of Bell's palsy. By addressing critical gaps in our understanding and fostering collaborative partnerships, we can work to improve outcomes and people's overall health. This disease is very serious.
